# Evaluation of cancer risk in patients with periodontal diseases

**DOI:** 10.3906/sag-1812-8

**Published:** 2019-06-18

**Authors:** Deniz Can GÜVEN, Ömer DİZDAR, Abdullah Cevdet AKMAN, Ezel BERKER, Emre YEKEDÜZ, Furkan CEYLAN, Batuhan BAŞPINAR, Ilgın AKBIYIK, Burak Yasin AKTAŞ, Deniz YÜCE, Mustafa ERMAN, Mutlu HAYRAN

**Affiliations:** 1 Department of Medical Oncology, Hacettepe University Cancer Institute, Ankara Turkey; 2 Department of Preventive Oncology, Hacettepe University Cancer Institute, Ankara Turkey; 3 Department of Periodontology, Faculty of Dentistry, Hacettepe University, Ankara Turkey; 4 Department of Medical Oncology, Faculty of Medicine, Ankara University, Ankara Turkey; 5 Department of Internal Medicine, Faculty of Medicine, Hacettepe University, Ankara Turkey

**Keywords:** Breast cancer, cancer risk, periodontal disease, prostate cancer

## Abstract

**Background/aim:**

In this study, we aimed to assess the cancer risk among patients with periodontal disease.

**Materials and methods:**

Patients diagnosed with periodontal diseases at Hacettepe University between 2007 and 2012 were included and data on the diagnosis of any cancer after periodontal disease were collected from patient files. The age- and sex-standardized incidence rates (SIRs) were calculated using Turkish National Cancer Registry 2013 data.

**Results:**

A total of 5199 patients were included. Median follow-up was 7.2 years. Patients with periodontal diseases had 17% increased risk of cancer compared with the expected counts for the corresponding age and sex groups (SIR: 1.17; 95% CI: 1.04–1.3, P = 0.006). The increased cancer risk was statistically significant in women (SIR: 1.24; 95% CI: 1.05–1.45, P = 0.008) but not in men. Among women with periodontal disease, the risks of breast cancer (SIR: 2.19) and head and neck cancer (SIR: 4.71) were significantly increased. Among men, the risks of prostate cancer (SIR: 1.84), head and neck cancer (SIR: 3.55), and hematological cancers (SIR: 1.76) were significantly increased.

**Conclusion:**

This study showed that periodontal diseases were associated with increased risk of several cancers. Besides other well-known benefits for health, the provision of oral/dental health should be considered and employed as a cancer prevention measure.

## 1. Introduction

Periodontal diseases are polymicrobial chronic inflammatory diseases that cause the damage of the periodontal ligaments and collapse of the adjacent alveolar bone. Periodontal diseases have many stages ranging from mild and short-lived gingivitis to severe periodontitis, which develops after persistent inflammation [1]. The worldwide prevalence of periodontal disease can be up to 90% and gingivitis affects almost half of the adult population [2].

It is thought that periodontal diseases may cause local inflammation as well as important systemic diseases in distant organs including cancers. Proposed mechanisms for this association include the bacteremia secondary to the weakened periodontal epithelium and systemic immune dysregulation [3,4]. The dysbiotic microenvironment in periodontal diseases is stated to create both an immune-evasive and a proinflammatory state, which is required for its persistence [3]. The role of systemic inflammation in increased cancer risk was supported by multiple human studies by the demonstration of increased circulatory cytokines and chemokines in periodontal diseases [5,6].

Several epidemiologic studies have shown that periodontal diseases are associated with an increased risk of cancers including, but not limited to, breast, lung, prostate, and hematological cancers [7–10]. However, assessment methods for periodontal disease in large epidemiological studies were heterogeneous as most studies utilized self-reported data [11] or administrative data [12] rather than examination by a periodontist. We previously showed that moderate to severe periodontitis diagnosed by a periodontist was associated with a 77% increase in cancer risk [13]. This high risk prompted us to further investigate cancer risk in a larger population of patients with any periodontal disease, including milder forms of periodontitis, gingivitis, etc. We performed this study to examine cancer risk in a large cohort of over 5000 patients with periodontal disease in comparison with the data of the Turkish National Cancer Registry (TNCR) in the same age and sex groups [14].

## 2. Materials and methods

### 2.1. Patients

This study was performed in the Hacettepe University Dentistry and Oncology Hospitals in Ankara, Turkey. Patients diagnosed with any periodontal disease in the Hacettepe University Oral Diagnosis Department and referred to the Periodontology Department between 2007 and 2012 were identified from the hospital registry. Data on the diagnosis of any cancer after periodontal disease were collected from patient files and oncology registries where available. Patients younger than 50 years of age and those with a prior cancer diagnosis were excluded. TNCR 2013 data were used for comparison of age- and sex-specific incidence rates.

The patients were diagnosed using standard clinical and radiographic parameters in accordance with the Periodontal Disease Classification System of the American Academy of Periodontology [15]. 

The study was approved by the Ethics Committee of Hacettepe University on 24.08.2017 with approval number 17/708.

### 2.2. Statistical analysis

Standardized incidence rates were calculated after adjustment for age and sex and compared with age and sex-specific incidence rates (SIRs) abstracted from the 2013 TNCR data [14]. The observed number of cases is based on the number of individuals diagnosed with cancer upon follow-up after the diagnosis of periodontal disease. Expected cases represent the total number of patients that would have been reported to the cancer registry within the same period of follow-up as per the TNCR rates under the null hypothesis of no increased risk, given the age and sex structure. The SIR and the 95% confidence interval (CI) for the SIR were calculated using OpenEpi version 3.01 software. A ratio greater than 1.00 indicates that there were more cases observed than expected. P < 0.05 was considered statistically significant.

## 3. Results

A total of 5199 patients with periodontal disease were included in the study. The median age was 57.7 years and 59% of the patients were female. Median follow-up was calculated as 7.2 years. Three hundred and nineteen new cancer cases were observed in follow-up. The most common cancers were breast cancer (67 of 153 cases) in women and prostate cancer (40 of 166 cases) in men.

The SIRs with 95% CIs are shown in Table 1. Patients with periodontal disease had 17% increased cancer risk (SIR: 1.17, 95% CI: 1.04–1.30, P = 0.006) compared to TNCR data for similar age and sex groups. The overall increased cancer risk did not reach statistical significance in men (SIR: 1.11, 95% CI: 0.95–1.28, P = 0.20), while in women with periodontal disease increased cancer risk was statistically significant (SIR: 1.24, 95% CI: 1.05–1.45, P = 0.008).

**Table 1:** Standardized incidence ratios (SIRs) with 95% confidence intervals for all and specific cancers in patients with periodontal disease.

All	5199	319	273.8	1.17	1.04–1.30	0.006
Male total	2151	166	150.2	1.11	0.95–1.28	0.197
Male prostate		40	21.7	1.84	1.34–2.49	<0.001
Male lung		23	38.3	0.6	0.39–0.89	0.013
Male head and neck		11	3.1	3.55	1.87–6.17	<0.001
Male hematologic		13	7.4	1.76	0.97–2.93	0.039
Male colorectal		14	14.2	0.99	0.56–1.62	0.957
Male bladder		13	12.3	1.06	0.59–1.77	0.842
Male stomach		7	9.2	0.76	0.33–1.50	0.468
Male pancreas		4	2	2	0.64–4.82	0.157
Female total	3048	153	123.6	1.24	1.05–1.45	0.008
Female breast		67	30.6	2.19	1.71–2.77	<0.001
Female lung		9	7.9	1.14	0.56–2.10	0.695
Female head and neck		8	1.7	4.71	2.19–8.93	<0.001
Female hematologic		8	7.4	1.08	0.5–2.05	0.825
Female colorectal		8	11.2	0.71	0.33–1.36	0.339
Female bladder		3	2.1	1.43	0.36–3.90	0.535
Female stomach		1	4	0.25	0.01–1.23	0.134
Female pancreas		3	2.8	1.07	0.27–2.92	0.905

In women with periodontal disease, breast cancer (SIR: 2.19, 95% CI: 1.71–2.77, P < 0.001) and head and neck cancer risks (SIR: 4.71, 95% CI: 2.19–8.93, P < 0.001) were significantly increased. In males, prostate cancer (SIR: 1.84, 95% CI: 1.34–2.49, P < 0.001), head and neck cancer (SIR: 3.55, 95% CI 1.87–6.17, P < 0.001), and hematological cancer risks (SIR: 1.76, 95% CI: 0.97–2.93, P = 0.039) were found to be higher than the general population data of similar age and sex groups (Table 1).

## 4. Discussion

In this study, we showed that the presence of any periodontal disease increased the risk of cancer by 17% in patients from a comprehensive dentistry hospital. The association was particularly significant for breast and head and neck cancer in women and prostate, head and neck, and hematological cancers in men. These findings are in accordance with the findings obtained in our previous study [13] and showed that in a population of patients with milder forms of periodontal disease, cancer risk is still increased, but the magnitude of the risk is lower than the risk for those with moderate to severe periodontitis (Table 2). 

**Table 2:** Comparison of standardized incidence ratios (SIR) with 95% confidence intervals for all and specific cancers in our two studies.

	Patient population	
	Any periodontal disease (current study) SIR (95% CI)	Moderate-severe periodontitis [13] SIR (95% CI)
All	1.17 (1.04–1.3)	1.77 (1.17–2.58)
Male total	1.11 (0.95–1.28)	1.69 (0.92–2.89)
Male prostate	1.84 (1.34–2.49)	3.75 (0.95–10.21)
Male hematological	1.76 (0.97–2.93)	6.97 (1.77–18.98)
Female total	1.24 (1.05–1.45)	1.84 (1.02–3.07)
Female breast	2.19 (1.71–2.77)	2.40 (0.88–5.33)
N	5199	280

A number of underlying mechanisms were proposed for the association between periodontal disease and cancer. These include systemic inflammation induced by the periodontal disease, immune dysregulation, and alteration of the oral flora [16–18]. Periodontitis was proposed to be associated with subclinical systemic inflammation [3,19]. Similar associations have been known for various chronic inflammatory disorders such as inflammatory bowel disease and colorectal cancer [3]. The increase in systemic markers of inflammation such as C-reactive protein, interleukin 6, and tumor necrosis factor-α in the plasma of periodontitis patients supports this association [5,6]. Increased levels of myeloperoxidase and superoxide dismutase, which are among the main regulators of inflammation, were found in periodontitis [20]. An increased gastric cancer risk due to increased inflammation in periodontitis was also proposed [21]. These findings led to the notion that immediate treatment of periodontal disease may reduce the inflammation and its remote effects on other organs [19]. Although mechanisms are unclear, periodontal disease treatment reduced the subsequent cancer risk by 28% in one study [22], which may be partly explained by a reduction in inflammation.

The highly vascular and fragile structure of the gingival tissue makes it a vulnerable entry site for oral pathogens during daily activities like eating and tooth brushing [6]. Carcinogenic byproducts of oral bacteria metabolism are suggested to be important in the link between periodontal disease and cancer [19]. For example, increased salivary acetaldehyde levels in poor dental health have been shown [23] and may be related to the mediation of oral microbiome contributions to cancer risk in periodontal disease. Oral microbiome changes have been reported in oral cavity cancers [24] and may be related to increased head and neck cancer risk in periodontitis patients. Periodontal pathogens also seem to play roles in the carcinogenesis of distant organs. Increased levels of *Fusobacterium nucleatum* and *Porphyromonas gingivalis* in the tumor tissue and feces of colorectal cancer patients and increased levels of *Porphyromonas gingivalis* in tumor tissue of esophagus cancer patients have been shown [25–27], but routes of dissemination and pathogenetic roles are yet to be determined. 

Numerous wide-scale epidemiological studies reported the association between periodontal disease and cancer risk. Periodontal diseases were found to be associated with a 14% increased cancer risk in the WHI cohort [10]. A cohort study from Taiwan that included more than 40,000 patients with chronic periodontitis showed a 23% increase in total cancer risk compared with age-matched controls in 5-year follow-up. A lower 5-year cancer-free survival in the chronic periodontitis cohort was also an interesting finding indicating a prognostic role of chronic periodontitis in cancer [28]. In a study by Michaud et al., a 13% increased risk of total cancer was observed among males who never smoked with periodontal disease. In this study, only smoking-related cancers appeared to increase. This finding suggested that alterations of immune pathways may play an important role in the mediation of periodontal disease and cancer association, giving the effects of smoking on altered immune response [8]. 

Breast cancer risk was increased more than twofold in patients with periodontal disease in this study, similar to our previous study but somewhat higher than in previous studies in the literature. In a study by Freudenhelm et al., in the WHI cohort, 73,737 women were followed for 6.7 years and the presence of periodontal disease was associated with 14% increased breast cancer risk [11]. Similarly, in a study from Taiwan, chronic periodontitis was associated with 23% increased breast cancer risk [28]. In the NHANES I follow-up study, the presence of periodontitis was associated with a 32% increased risk of breast cancer but gingivitis did not show an increased risk [29]. In a study by Söber et al., the presence of any missing molars in the mandible was associated with increased breast cancer risk (odds ratio (OR) of 2.36) [30]. The latter two studies also showed higher risks of cancer in patients with more advanced periodontal disease resulting in tooth loss. Our lack of radiological data precluded such a stratification in our study.

There are conflicting data in the literature on the link between prostate cancer and periodontal disease. Michaud et al. reported an inverse association between tooth loss and prostate cancer in a male health professionals cohort but did not include nonaggressive prostate cancer in the analyses [9]. Hujoel and Lee reported increased prostate cancer risks with periodontitis but with rather different magnitudes (81% vs. 14%) [7,29]. Prostate cancer risk was increased by 84% in our study and this finding may be partly explained by our inclusion of all prostate cancer cases in the analyses. Differences in the gut microbiome of prostate cancer patients and patients with benign prostatic conditions were reported [31]. Estemalik et al. showed oral pathogens in patients with chronic prostatitis and benign prostatic hyperplasia [32]. These findings indicate a possible association between the oral microbiome and prostate diseases, but comments on driver or bystander effects could not be made without further data [33].

Periodontal diseases are consistently associated with an increased risk of head and neck cancers, not surprisingly when considering the role of microbiome changes and chronic inflammation in both conditions [19]. Like periodontal diseases, tooth loss alone was also associated with head and neck cancer risk [34]. In a metaanalysis of studies published before March 2013, periodontal disease was associated with an OR of 2.63 for head and neck cancers. Although smoking and alcohol are among the most common risk factors for both periodontal disease and head and neck cancer, in the studies covaried for smoking and alcohol, periodontal disease still remained an independent risk factor with an OR of 2.23 [35]. Our findings are consistent with the previous literature and showed a 3- to 4-fold increased cancer risk in both males and females in a heterogeneous population of patients with various periodontal diseases.

There are limited data on the literature regarding the link between hematological cancers and periodontal disease. Michaud et al. reported a 30% increase in hematological cancer risk in periodontal disease in a study of male health professionals [9]. In the Health Professionals Follow-Up Study, periodontal disease was associated with increased non-Hodgkin lymphoma and chronic lymphocytic leukemia/small lymphocytic lymphoma risks [36]. Hiraki et al. reported no significant association with periodontal disease and hematological cancers [37]. Hematological cancers were increased in males in both of our studies, but due to the low number of cases, SIR values for hematological cancer subtypes were not calculated.

The link between lung cancer and periodontal disease was investigated several times in the literature [9,37–39]. After adjustment for smoking, some studies did not find an independent association between periodontal diseases and lung cancer [29,38], which may be due to overadjustment in regard to healthy behavior patterns. There was an inverse association between periodontal disease and lung cancer in men, which was an unexpected finding. Due to lack of adjustment for smoking in our study, we think that underreporting may be the main reason for the less frequent occurrence of lung cancer in men with periodontal disease.

Strengths of our study include the relatively large sample size and diagnosis of periodontal disease by an experienced team. Confirmation of cancer diagnoses from the pathology reports rather than administrative data was important for the reliability of results. However, our study is subject to a number of limitations. First of all, cancer data were taken from patients’ files and hospital records; thus, cancers diagnosed in other centers may have been underreported, altering the results biased towards the null. In addition, the severity and generality of periodontal disease were not evaluated. This prevented us from making further comments on the association of specific periodontal disease parameters and cancer risk. Another limitation was the inability to perform adjustments for factors such as smoking, socioeconomic status, diet, and comorbidities due to lack of data for most patients. The absence of family histories may also be considered among limitations due to possible shared susceptibility driving both conditions, although this shared risk is most evident in the aggressive periodontitis that occurs in early ages [40]. Our study only included patients older than 50 years, which lessens the possibility of confounding due to genetic susceptibility.

Routine clinical applications regarding periodontal disease and cancer risk have yet to be defined. Periodontal disease per se might have a role in carcinogenesis or it might simply be a consequence of an unhealthy lifestyle and habits [41]. Prospective studies with long-term follow-up may aid in discrimination in the future.

In conclusion, these data suggest that chronic inflammatory periodontal diseases are not only a health problem that affects the oral cavity but also has the potential to increase the risk of cancer in local and remote organs by microbiological and immunological mechanisms. Besides other well-known health benefits, maintaining oral/dental health should also be considered and employed as a cancer prevention measure.
